# Lifestyle Factors Influencing Dietary Patterns of University Professors

**DOI:** 10.3390/ijerph18189777

**Published:** 2021-09-16

**Authors:** María López-Olivares, Carlos De Teresa Galván, Teresa Nestares, Elisabet Fernández-Gómez, Carmen Enrique-Mirón

**Affiliations:** 1 Doctoral Degree School, Melilla Campus, University of Granada, Calle Santander s/n, 52001 Melilla, Spain; marialopez93@correo.ugr.es; 2 Andalusian Sport Medicine Centre, 18007 Granada, Spain; cdeteresa2403@gmail.com; 3 Department of Physiology, Faculty of Pharmacy, University of Granada, 18071 Granada, Spain; nestares@ugr.es; 4 Biomedical Research Centre (CIBM), Institute of Nutrition and Food Technology “José MataixVerdú” (INYTA), University of Granada, 18071 Granada, Spain; 5 Department of Nursing, Faculty of Health Sciences, Melilla Campus, University of Granada, Calle Santander s/n, 52001 Melilla, Spain; 6 HUM-613 Research Group, Department of Inorganic Chemistry, Faculty of Health Sciences, Melilla Campus, University of Granada, Calle Santander s/n, 52001 Melilla, Spain; cenrique@ugr.es

**Keywords:** Mediterranean pattern, western pattern, professors, university, sociodemographic factors, healthy lifestyle

## Abstract

The objectives of this study are to identify eating patterns of university professors and to assess the relationships among sociodemographic factors in relation to lifestyle and physical activity. It is a cross-sectional, descriptive-correlational, and observational study with a representative sample of 127 educators, which covers almost the total population of university professors belonging to one of the campuses of the University of Granada (Spain). Two eating patterns were identified a posteriori through explanatory factor analysis: a Western pattern characterised by the consumption of dairy products, eggs, meat, sausages, refined oils, and butter, sugar, processed baked goods, and sugar-containing beverages and alcoholic drinks, and a Mediterranean pattern based on olive oil, fish, fruits, nuts, vegetables, pulses, cereals, and honey, which explain the 20.102 and 17.411 of variance, respectively. Significant differences are observed between the two genders with respect to anthropometric characteristics (weight and size, *p* < 0.001 in both cases) and to nutritional status (*p* = 0.011). Origin (*p* = 0.022) and level of physical activity (*p* = 0.010) were significantly related to adherence to a Western diet pattern. In the case of the Mediterranean diet pattern, significant differences are observed according to the professors’ type of bachelor’s degree (*p* = 0.37). This study provides evidence on factors having an impact on adherence to eating patterns of professors of the University of Granada, and it suggests that programmes addressed to such groups should be developed to promote health.

## 1. Introduction

University professors require optimal health conditions to fulfil their main tasks such as research, university management, continuous improvement of teaching since these are essential elements in the educational process of future professionals [1,2,3,4]. Professors are a group of professionals who are extremely important for society and who are subject to factors of stress, such as long working days, a decrease in the time devoted to leisure and sport, having little time to rest, lack of sleep, and also to the manner in which they nourish themselves [5]. Physical activity, health status, and nutritional habits are important factors to be taken into account for a healthy lifestyle [6] since they seem to have an influence on cellular ageing [7,8], besides reducing the risk of cardiovascular events [9]. A sedentary lifestyle and consumption of a high-calorie diet constitute one of the biggest problems in modern society, due to their relationship with excessive fat accumulation in the body, which leads to overweight or obesity [10,11], which data have been reflected in studies in which more than half the professors suffered from overweight or obesity [12,13].

Eleven million deaths worldwide, 22% of all the deaths among adults, were attributable to dietary risk factors, based on the Global Burden of Disease Study 2017 [14]. Unhealthy eating habits are associated with a higher prevalence of chronic noncommunicable diseases (CNCDs) and with higher mortality both in developed countries and in developing countries [14]. Eating behaviours and unhealthy lifestyles associated with sedentarism (spending much time sitting and practising no physical activity) have their origin in childhood and remain in adult life [15,16,17].

A healthy diet is one including macronutrients in adequate proportions to satisfy energy and physiological needs. The Mediterranean diet has been selected by the FAO as a model for the assessment of dietary sustainability [18]. It has been scientifically well characterised and recognised as a healthy eating pattern. That is why a higher adherence to the Mediterranean diet has been widely associated with significant improvements in health [19,20]. However, the ANIBES study carried out in Spain in 2015 stated that a high percentage of the Spanish population did not follow such eating pattern and consumed high intakes of saturated fatty acids (SFAs) and sugars and did not achieve important nutritional requirements such as fibre, calcium, zinc, folic acid, and vitamins A and C [21].

Among the most significant changes in the epidemiological and nutritional profiles over the last decades [22], it is worth highlighting an increase in consumption of industrial food and a decrease in the consumption of fruits and vegetables [23], which has a direct impact on an increase in the prevalence of chronic noncommunicable diseases [24]. Thus, the selection of daily food contributes to the risk of developing hypertension, hypercholesterolemia, overweight or obesity, and inflammation, including cardiovascular diseases, diabetes, and cancer [25]. In fact, an increase in chronic noncommunicable diseases is related to eating patterns that are westernising more and more [26], characterised by high levels of fatty processed meats, saturated fat, refined grains, salt, and sugars, but that lack fresh food, fruits and vegetables [27].

That is why the impact of general eating patterns is more and more important, instead of the isolated intake of nutrients due to the importance thereof for metabolic health [28]. In general, the eating patterns study is a more complete approach that has proven to be useful to provide significant results to a determined population [29,30]. Eating patterns represent the general combination of food usually consumed, which as a whole has synergistic effects on health. Beneficial eating patterns reported by scientific evidence share several key characteristics [28,31,32]. These include minimally processed foods, such as fruits, walnuts/seeds, vegetables, pulses, whole grains, fish, yoghurt, and vegetable oils, and less red meat, processed meat, refined grains, starch, and added sugars. The Mediterranean pattern is one of the most frequent eating patterns mentioned in scientific literature due to its healthy characteristics, as already described. Its strong and consistent association with health benefits [33], particularly in the case of cardiovascular diseases, has been evidenced in randomised controlled trials [34,35] and observational studies [36,37,38,39].

The main objective of this study is to determine, by means of posteriori techniques, the main eating patterns of the teaching and research staff (TRS) of the Melilla campus of the University of Granada and the association thereof with different groups of foods as well as with several sociodemographic factors and other variables such as the level of physical activity done.

## 2. Materials and Methods

### 2.1. Study Design and Participants

Cross-sectional, descriptive-correlational, and observational study with a representative sample of university professors of the Melilla campus of the University of Granada. The target population is formed by teaching and research staff of the three faculties constituting said campus (Faculty of Health Sciences, Faculty of Social and Legal Sciences, and Faculty of Educational and Sport Sciences), which, in the 2019/2020 academic year, amounted to 185 professors, the participating sample (totally random) being of 127 professors (62 men and 65 women), aged 29–67 years. Inclusion criteria for participants have included being a full-time professor at the Melilla campus (University of Granada), acceptance of the study by the respondent, and signing informed consent. On the other hand, professors with no full-time relationship with the university and those not signing the informed consent who, therefore, did not want to participate in this study have been excluded.

The recruitment was made face to face in the department of each professor, and the researchers obtained consent for participation from the university professors of the Melilla campus of the University of Granada.

#### 2.1.1. Sociodemographic Data

Sociodemographic data corresponding to age, sex, faculty, origin, marital status, and professional stability were collected through a self-administered questionnaire during the data collection period; age (years old), sex (woman/man), faculty (Health Sciences/Education Sciences/Social and Legal Sciences), Origin (Melilla/rest of Spain), marital status (single/married/widowed/cohabitating/separated/divorced/other), professional stability (yes/no).

#### 2.1.2. Anthropometric Analysis

For the weight and height measures, the protocol stated by the World Health Organisation (WHO) was followed [40]. Weight data were collected using a bioimpedance metre (inBody R20) that incorporates 8 tactile electrodes to avoid the possibility of error or inaccuracies. Measurement was performed with fasting for 8 h or more and with the bladder emptied before the assessment. Height was measured using a mechanical telescopic height measuring rod (SECA 222) with a measurement range of 6–230 cm and an accuracy of 1 mm. From the weight and height, the BMI was obtained, which variable allowed the classification of participants in four categories: underweight (BMI < 18.5 kg/m^2^); normal weight (BMI = 18.5–24.9 kg/m^2^); overweight (BMI = 25.0–29.9 kg/m^2^); obesity (BMI ≥ 30.0 kg/m^2^), according to the criteria established by the Spanish Society for the Study of Obesity (SEEDO) [41].

#### 2.1.3. Physical Activity Analysis

Physical activity was determined by means of the short version of the International Physical Activity Questionnaire (IPAQ) [42]. The time devoted to vigorous and moderate activities, walking and sitting during the last week was considered. Data obtained were treated following the protocol established in the Guidelines for Data Processing and Analysis of the IPAQ [43].

#### 2.1.4. Food Consumption Analysis

Participants were personally assisted by a qualified professional in order to complete a validated food frequency questionnaire (FFQ) provided by the Autonomous Government of Andalusia [44]. The questionnaire collects data on times per day, per week, and per month when food and drinks included in the questionnaire were consumed, such amounting to 136 foods gathered in 9 groups: dairy products, eggs, meat and fish, vegetables, fruits, pulses and cereals, oils and fat, beverages, bakery and pastries, and miscellaneous. The FFQ assesses participants’ diet during the last 12 months.

#### 2.1.5. Statistical Analysis

Data were analysed by using the statistical programme SPSS 24.0 (International Business Machines Corporation (IBM), Armonk, NY, USA). To calculate significant differences in prevalence, Pearson’s chi-square test was used, or Fisher’s exact test was used when variables were nominal, and Kendall’s tau-b was used when variables were ordinal. Differences among medians were assessed using the Mann–Whitney U test.

A factor analysis was conducted (principal component analysis, PCA) to identify eating patterns by using the average weight consumed (g/day) by each person out of 20 groups of foods. Two factors explaining the 20.1% and 17.4% of the total variance, respectively, were selected. Factor 1 (called the Western eating pattern) is positively associated with the consumption of dairy and dairy-derived products, eggs, meat, sausages, refined oils and butter, sugar, processed baked goods, snacks, and sugar-containing beverages and alcoholic drinks. Factor 2 (Mediterranean eating pattern) is positively related to the consumption of olive oil, fish, fruits, nuts, vegetables, pulses, cereals, honey, to a lesser degree than the consumption of dairy and dairy-derived products, meat and sausages, and negatively to the consumption of sugar-containing beverages. In order to verify the suitability of the factor analysis, Bartlett’s sphericity coefficient and Kaiser-Meyer-Olkin (KMO) test were employed. To assess the degree of correlation among variables, a KMO value > 0.60 was adopted. An orthogonal rotation (Varimax) was applied to the factor burden matrix to optimise the correlation between foods and factors and to facilitate their interpretation. The number of factors to retain was determined according to the sediment graph, the proportion of variance explained, and their interpretability. The denomination of each eating pattern was established, taking into account which foods were sufficiently correlated to each factor (burden factor ≥ 0.200).

Every subject obtained a score calculated as the sum of consumption in each group of food weighted by the pertinent factor in PCA. Scoring coefficients in each factor were estimated by using the Anderson-Rubin [45] method, which produces scores not correlated to a median of 0 and a standard deviation of 1. A higher score indicates higher adherence to the factor. This factor analysis was conducted for each group of food, both with a Z-score calculation and without such calculation. The accepted significance level in every statistical trial was *p* < 0.05.

#### 2.1.6. Ethical Aspects

This study has been conducted following the directives established by the Declaration of Helsinki. Every participant signed the pertinent informed consent.

## 3. Results

Table 1 shows the sociodemographic characteristics of university professors of the Melilla campus. The median age for the total sample was of 47.28 ± 11.36 years, reflecting significant differences between both genders (*p* = 0.038).

Factors related to lifestyle are collected in Table 2. As regards weight, size, and body mass index (BMI), significant differences between genders are observed, the median values being higher in men as compared to women. Likewise, significant differences are found between genders in connection to nutritional status (*p* = 0.011). Among professors, 40.2% of them are overweight (53.2% of men and 27.7% of women), and 10.2% suffer from obesity (11.3% of men and 9.2% of women). In general, 50.4% of the sample shows an excess of body weight (overweight and obesity), significant differences being found between genders (64.5% of men and 36.9% of women). Finally, 1.6% of the sample shows underweight, all cases being observed in women. The percentage of university professors who do high-intensity physical activity is 42.5%, while the percentage of subjects doing moderate- and low-intensity physical activity is 32.3% and 25.2%, respectively. Men and women show no significant differences regarding the level of physical activity done.

Table 3 shows eating patterns determined according to food groups related to daily consumption.

**Figure 1:**
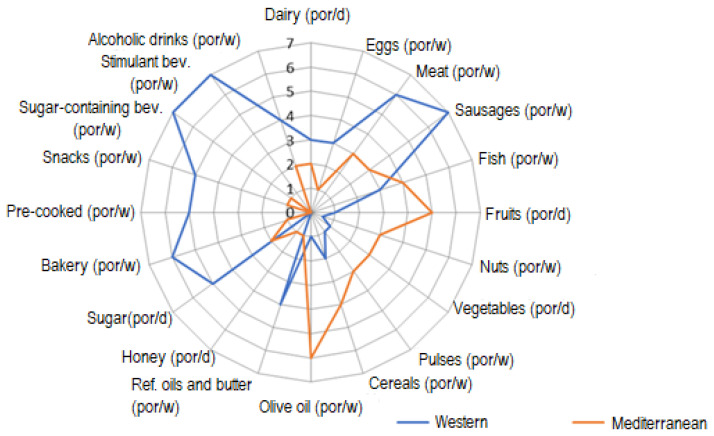
Medians of consumption of different food groups (portions/day or portions/week) according to the scores reached in the Western pattern (Factor 1) and Mediterranean pattern (Factor 2).

Figure 1 represents the medians of consumption portions (expressed per day or per week, pursuant to the recommendations for the Spanish population) of the different food groups for certain dietary patterns.

The distribution of university professors according to their adherence to Western and Mediterranean diet patterns (tertiles), sex, bachelor’s degree, nutritional status, and physical activity level is reflected in Table 4 and Table 5. From the associations studied, for the Western pattern, significant differences are observed as regards origin (*p* = 0.022) and physical activity level (*p* = 0.010). In the Mediterranean pattern case, significant differences are only observed with respect to the centre where they teach (*p* = 0.37).

## 4. Discussion

In this study, based on the data on a representative sample of university professors of the Melilla campus, two eating patterns were identified. On the one hand, the Western pattern, characterised by high consumption of dairy and dairy-derived products, eggs, meat, sausages, refined oils and butter, sugar, processed baked goods, snacks and sugar-containing beverages, and alcoholic drinks. On the other hand, the Mediterranean eating pattern, which was positively related to the consumption of olive oil, fish, fruits, nuts, vegetables, pulses, cereals, honey, to a lesser degree to the consumption of dairy and dairy-derived products, meat, and sausages, and negatively, to the consumption of sugar-containing beverages. By means of the ordinal analysis, sociodemographic and lifestyle-related determinants were established. With respect to the centre where professors teach, statistically significant differences were observed between both genders; in addition, men show a higher BMI than women, significant differences being found between both genders, which results are similar to those found in a study conducted by Bacârea et al. [46].

Eating pattern approaches are more frequently used to assess diet quality as a whole. In the first place, it is necessary to determine the level of compliance with eating guidelines to define a diet before predefined eating patterns are assessed as favourable or unhealthy. Several recent studies associate certain factors with what is known as dietary patterns [47,48]. Patterns obtained in this study were similar to those collected by Roger et al. [49], Agodi et al. [50], and Brigham et al. [51] in their studies on eating patterns and associated diseases. This approach may improve the limitations of conventional methods to study individual foods [52,53,54] or groups of food, thus allowing the analysis of eating patterns [52,53,55] and interpreting eating behaviour that may be used to establish public health recommendations [52,53].

In this study, the explanatory factor analysis and principal component analysis were used, two techniques frequently used to create a posteriori eating patterns [56,57,58,59,60,61]. Some of the most used tools to collect data on eating patterns are dietary records, 24 h reminders, and food consumption frequency (FFQ) [57]. FFQ is generally used for the creation of eating patterns [56], apart from being one of the most appropriate instruments of eating assessment in large epidemiological studies, since it provides information on participants’ usual diet for a longer period of time [58,59].

Higher adherence to the Western eating pattern has been related to a decrease in physical activity according to Ciprián et al. [60] in their study on Mediterranean and Western eating patterns in the adult population, data that are very similar to that obtained in this study. Furthermore, the Western eating pattern has been associated with weight gain [50], which may explain the reason why more than half of our sample suffers from overweight or obesity. Likewise, this pattern is linked to an increase in the obesity rate [62,63,64], given that it shows the prevalence of food such as bakery, snacks, salt and sugars, and energy-dense, nutritionally unbalanced refined oils [65,66]. Considering that 50.4% of university professors suffer from overweight or obesity, the adoption of such Western pattern by part of the sample could be the reason for such excess of fat, so that the need to implement programmes to promote health addressed to professors is confirmed, even more when the Western pattern shows a higher risk of metabolic syndrome (MetS), cardiovascular and coronary heart disease [67], pulmonary disease [51], diabetes mellitus [68], cognitive impairment and dementia [69,70], which risk is reduced among the population following a healthy eating pattern such as the Mediterranean [67,71].

With respect to the Mediterranean pattern, it is professors at the Faculty of Social and Legal Sciences who show a higher adherence to it, which is a curious result taking into account that it is healthcare-areas professors the ones showing a higher adherence to such pattern [72]. This study on the distribution of eating patterns observed in a sample of the adult population in the autonomous city of Melilla is particularly interesting since it is a city on the Mediterranean. The Mediterranean pattern includes a wide variety of foods, the emphasis being placed on the preparation of tasty foods, moderately accompanied by red wine, social relations, stress reduction together with regular physical activity, accompanied by suitable weather [73,74]. However, even when such a city has the suitable characteristics to adopt a Mediterranean pattern adequately, it may be noted that the individuals from the very own city show a higher adherence to the Western pattern, as opposed to the rest of Spain.

It is worth noting that among the university population, most studies are addressed to students, the ones conducted with professors being scarce. Among students, research conducted in different Spanish regions shows a medium/low adherence to the Mediterranean pattern [75,76,77,78], regardless of physical activity [79,80], which has an impact on the need to promote healthier lifestyles among the university population in general.

One of the strengths of this study is that every data of the sample was gathered personally by a qualified nutritionist to guarantee their accuracy. Moreover, although there are several investigations addressing eating patterns, this study is one of the first studies to assess such patterns among professors from different faculties, a group of professionals extremely important for society, subject to stress factors, with little time for leisure and sport, and little time to rest, which may affect the way they nourish themselves [5]. Professors play a key role in conveying to students of knowledge and skills that are essential to adopt responsible health decisions. According to Montenegro et al. [81], in their study on the assessment of nutritional education intervention in professors and students, the performance of such a group has improved the knowledge on and intake of healthy foods. It is also worth noting the geographical area in which the city of Melilla is located, surrounded by the Mediterranean Sea and gathering all the characteristics to adopt this pattern in an adequate manner; nevertheless, pursuant to our results, said characteristics are not sufficient for a part of our sample, which represents that they have adopted a Western pattern.

The results of this study must be interpreted, taking some limitations into account. Firstly, the sample is representative considering the population to which it is addressed, but further research is required to investigate such associations in a general sample of university professors. Secondly, since it is a cross-sectional study, it only represents a description of the current situation of such a group, thus not being possible to establish a cause-effect relationship. On the other hand, given that diet represents a complex variable that varies depending on the region, eating patterns may demonstrate variations preventing comparison among countries and continents. Most part of the literature assessing the effects of eating patterns is based on populations outside Spain, which is why further research is required within Spain to continue studying this topic. Likewise, it is worth noting that existing evidence in such a population is limited so that it would be necessary to continue studying this topic.

## 5. Conclusions

Following a recommended and healthy eating pattern by university professors may be deemed a key factor in the improvement and promotion of health. Positively, most participants represent that they do physical activity; however, on the other hand, half the population suffers from overweight or obesity. Living in Melilla and the lack of physical activity have an impact on some professors’ Western eating patterns and, nevertheless, the type of bachelor’s degree has an influence on the adherence to the Mediterranean pattern. Therefore, it is necessary to design dietary guidelines that shall promote the modification of eating behaviour with the aim of improving university professors’ nutritional status, as well as to provide specific training where the most relevant healthy diet aspects shall be addressed in order to be able to study and evaluate results after such interventions.

## Figures and Tables

**Table 1:** Sociodemographic characteristics of university professors of the Melilla campus (University of Granada).

	Total (*n* = 127)	Male (*n* = 62)	Female (*n* = 65)	*p*
Age (years old)	46.0 (45.3–49.3)	49.5 (46.5–52.3)	44.0 (42.5–47.9)	0.038
Faculty				
Education and Sport Sciences	51 (40.2)	26 (41.9)	25 (38.5)	0.001
Health Sciences	36 (28.3)	9 (14.5)	27 (41.5)	
Legal and Social Sciences	40 (31.5)	27 (43.6)	13 (20.0)	
Origin				
Melilla	80 (63)	35 (56.5)	45 (69.2)	0.136
Rest of Spain and others	47 (37)	27 (43.5)	20 (30.8)	
Marital status				
Single/Separated/Divorced/Widowed	31 (24.4)	14 (22.6)	17 (26.2)	0.639
Married/Cohabitating	96 (75.6)	48 (77.4)	48 (73.8)	
Professional stability				
Yes	80 (63)	41 (66.1)	39 (60.0)	0.475
No	47 (37)	21 (33.9)	26 (40.0)	

Values express *n* (%). Statistically significant differences between both genders were analysed using Pearson’s chi-square (χ^2^) and Fischer’s exact test for comparison of proportions, and Mann–Whitney U test for comparison of medians.

**Table 2:** Anthropometric characteristics and lifestyle of university professors of Melilla campus (University of Granada).

		Total (*n* = 127)	Male (*n* = 62)	Female (*n* = 65)	*p*
Weight (kg)		72.3 (70.4–75.6)	82.1 (79.1–84.7)	62.4 (61.4–67.5)	<0.001
Height (cm)		169.0 (168.1–170.9)	173.0 (172.5–176.2)	165.0 (163.4–166.2)	<0.001
BMI (kg/m^2^)		25.0 (24.2–25.7)	25.4 (25.0–27.2)	22.6 (22.9–24.9)	0.004
Nutritional status (kg/m^2^)					
	Underweight (<18.5)	2 (1.6)	0	2 (3.1)	0.011
	Normal weight (18.5–24.9)	61 (48.0)	22 (35.5)	39 (60)	
	Overweight (25.0–29.9)	51 (40.2)	33 (53.2)	18 (27.7)	
	Obesity (>30)	13 (10.2)	7 (11.3)	6 (9.2)	
Physical activity done					
	Low intensity	32 (25.2)	15 (24.2)	17 (26.2)	0.840
	Moderate intensity	41 (32.3)	19 (30.6)	22 (33.8)	
	High intensity	54 (42.5)	28 (45.2)	26 (40.0)	

Values express medians (CI) and *n* (%). Statistically significant differences between genders were analysed by using Pearson’s chi-square (χ^2^) and Fischer’s exact test for the comparison of proportions, and Mann–Whitney U test for the comparison of medians.

**Table 3:** Eating patterns obtained on the basis of food groups related to daily consumption.

Food Group	FACTOR 1: Western	FACTOR 2: Mediterranean
Explained variance	20.102	17.411
Dairy and dairy-derived products	0.373	0.286
Eggs	0.343	-
Meat	0.790	0.372
Sausages	0.741	0.380
Fish	0.347	0.442
Fruits	-	0.723
Nuts	-	0.250
Vegetables	-	0.266
Pulses	-	0.321
Cereals	0.250	0.340
Olive oil	-	0.668
Refined oils and butter	0.371	-
Honey	-	0.282
Sugar	0.513	-
Bakery	0.610	-
Pre-cooked	0.488	-
Snacks	0.715	-
Sugar-containing beverages	0.488	−0.297
Stimulant drinks	-	-
Alcoholic drinks	0.613	-

**Table 4:** Distribution of university professors according to their adherence to the Western eating pattern (by tertiles), sex, centre, origin, marital status, professional stability, nutritional status, and physical activity.

			Eating Patterns			*p*
			T1	T2	T3	
Factor 1: Western	Sex					
		Male (*n* = 62)	21 (33.9)	19 (30.6)	22 (35.5)	0.801
		Female (*n* = 65)	21 (32.8)	24 (35.9)	20 (31.3)	
	Faculty					
		Education and Sport Sciences (*n* = 51)	19 (38.0)	17 (32.0)	15 (30.0)	0.620
		Health Sciences (*n* = 36)	10 (27.8)	15 (41.7)	11 (30.5)	
		Legal and Social Sciences (*n* = 40)	13 (32.5)	11 (27.5)	16 (40.0)	
	Origin					
		Melilla (*n* = 80)	21 (26.6)	33 (41.8)	26 (31.6)	0.022
		Rest of Spain and others (*n* = 47)	21 (44.7)	9 (19.1)	17 (36.2)	
	Cohabitation					
		Alone (*n* = 31)	13 (40.0)	7 (23.3)	11 (36.7)	0.399
		Living together (*n* = 96)	30 (31.3)	35 (36.5)	31 (32.2)	
	Professional stability					
		Yes (*n* = 80)	22 (26.6)	30 (38.0)	28 (35.4)	0.103
		No (*n* = 47)	21 (44.7)	12 (25.5)	14 (29.8)	
	Nutritional status					
		BMI < 25.0 (*n* = 63)	19 (30.2)	24 (38.1)	20 (31.7)	0.662
		BMI ≥ 25.0 (*n* = 64)	23 (35.9)	19 (29.7)	22 (34.4)	
	Physical activity					
		Low intensity (*n* = 32)	7 (22.6)	9 (25.8)	16 (51.6)	0.010
		Moderate intensity (*n* = 41)	10 (24.4)	20 (48.8)	11 (26.8)	
		High intensity (*n* = 54)	25 (46.3)	14 (25.9)	15 (27.8)	

T1, first tertile; T2, second tertile; T3, third tertile; BMI, body mass index. Values are expressed: *n* (%). Statistically significant differences among the different groups assessed were analysed using Pearson’s chi-square (χ^2^) and Kendall’s tau test.

**Table 5:** Distribution of university professors according to their adherence to the Mediterranean eating pattern (by tertiles), sex, centre, origin, marital status, professional stability, nutritional status, and physical activity.

			Eating Patterns			*p*
			T1	T2	T3	
Factor 2: Mediterranean	Sex					
		Male (*n* = 62)	17 (26.4)	20 (33.3)	25 (40.3)	0.383
		Female (*n* = 65)	25 (38.5)	19 (29.2)	21 (32.3)	
	Faculty					
		Education and Sport Sciences (*n* = 51)	15 (29.4)	17 (33.3)	19 (37.3)	0.037
		Health Sciences (*n* = 36)	16 (44.4)	14 (38.9)	6 (16.7)	
		Legal and Social Sciences (*n* = 40)	11 (27.5)	9 (22.5)	20 (50.0)	
	Origin					
		Melilla (*n* = 80)	26 (32.5)	26 (32.5)	28 (35.0)	0.988
		Rest of Spain and others (*n* = 47)	16 (34.0)	14 (29.8)	17 (36.2)	
	Cohabitation					
		Alone (*n* = 31)	10 (32.3)	9 (29.0)	12 (38.7)	0.974
		Living together (*n* = 96)	31 (32.3)	31 (32.3)	34 (35.5)	
	Professional stability					
		Yes (*n* = 80)	24 (30.4)	24 (30.4)	32 (39.2)	0.555
		No (*n* = 47)	17 (36.4)	16 (34.1)	14 (29.5)	
	Nutritional status					
		BMI < 25.0 (*n* = 63)	18 (28.6)	24 (38.1)	21 (33.3)	0.250
		BMI ≥ 25.0 (*n* = 64)	23 (35.9)	16 (25.0)	25 (39.1)	
	Physical activity					
		Low intensity (*n* = 32)	9 (28.1)	13 (40.6)	10 (31.3)	0.808
		Moderate intensity (*n* = 41)	12 (29.3)	13 (31.7)	16 (39.0)	
		High intensity (*n* = 54)	20 (37.0)	15 (27.8)	19 (35.2)	

T1, first tertile; T2, second tertile; T3, third tertile; BMI, body mass index. Values are expressed: *n* (%). Statistically significant differences among the different groups assessed were analysed using Pearson’s chi-square (χ^2^) and Kendall’s tau test.

## Data Availability

The data presented in this study are available on request from the corresponding autor.
